# The psychological impact of sexual torture: A gender-critical study of the perspective of UK-based clinicians and survivors

**DOI:** 10.1177/13634615221089491

**Published:** 2022-04-06

**Authors:** Roghieh Dehghan, Caroline Osella

**Affiliations:** 1 Centre for Gender and Global Health, 4919 University College London; 2 Sussex Asia Centre, 1948 University of Sussex

**Keywords:** body politics, gender, mental health, Muslims, refugees, sexual torture

## Abstract

Despite the high prevalence of sexual torture and its close link with gender, little work has been published on refugee torture survivors from Muslim-majority countries. The aim of this project was to introduce a gender-critical framework, that draws on post-modern and post-colonial feminism, to the study of sexual torture in terms of its operationalization and psychological impact in Iranian, Afghan, and Kurdish refugees in the United Kingdom (UK). This exploratory qualitative research was conducted in collaboration with two voluntary organizations in the UK. Mental healthcare providers (HCPs) were invited to participate through convenience sampling from amongst their staff as well as from community mental health services. Torture survivors were recruited through snowball sampling. The study consists of two parts: 1) semi-structured face-to-face interviews with a total of eight experts (doctors and therapists) and three torture survivors; followed by 2) a focus group with four experts to discuss the emerging results from the interviews and together reflect on the politics of gender and sexuality in the context of torture (‘assisted sense-making’). A thematic gender-critical analysis was performed for the qualitative data. Our findings from interviews with (only Kurdish) torture survivors and HCPs suggest that gender mediates the impact of sexual torture at the intersection of gender, cultural norms, forms of social inequality, and body politics. The conclusions of the study will have implications for health services by deepening our understanding of variables that intersect in an entangled and unpredictable network.

## Introduction

A high prevalence of psychological difficulties resulting from an array of pre-migratory trauma, including torture, has been reported in refugees (Duffy et al., 2017; Fazel et al., 2005). Estimates suggest that 30% to 84% of asylum seekers in high-income countries have been subjected to torture (Duffy et al., 2017; Kalt et al., 2013). Torture is defined by the United Nations Convention against Torture (CAT) (UN, 1984) as an act committed by state or state-like actors for specific purposes—such as punishment, intimidation, or obtaining information—that results in severe mental and physical suffering of the victim (United Nations, 1984). Correspondingly, sexual torture is ‘any sexual offence’ in the context of torture. The World Health Organization (WHO) defines ‘any sexual offence’ as conduct beyond rape and penetration, to include ‘unwanted sexual comments or advances’ as well as sexual humiliation and threats (Krug et al., 2002, p. 149).

Sexual torture is reported by 63–80% of female and 25–56% of male torture survivors (Busch et al., 2015; Lunde & Ortmann, 1990). These figures are consistent with the experience of tortured refugees from Muslim-majority countries. For instance, more than half of Iranian torture survivors (60%) examined at Freedom from Torture (FFT) (the largest charity for research, psychotherapy, and support of torture survivors in the United Kingdom (UK), which was founded in 1985) reported having been sexually violated (Freedom from Torture, 2013). For comparison, the rates were 30% for female and 1% for male Kurdish clients (presumably under-reported) (Bradley & Tawfiq, 2006). Despite this high prevalence, sexual torture in refugees from Muslim backgrounds has been poorly researched to date (Dehghan, 2018).

Torture can result in a wide range of physical problems, including scars, chronic pain, fatigue, insomnia, genitourinary complications, sexually transmitted infections, and unwanted pregnancies. Equally important, torture is also associated with a variety of emotional and mental health problems, such as personality changes, depression, anxiety, and post-traumatic stress disorder (PTSD) as well as self-harm and suicide (Dehghan, 2018; Isakson & Jurkovic, 2013).

Not surprisingly, there is evidence for a link between exposure to multiple traumas and the prevalence of PTSD in tortured refugees (Young & Chan, 2015, p. 26). Polytraumatization is more likely to occur among female refugees (Kastrup & Arcel, 2004). Ibrahim Kira et al.’s (2010) empirical study with 160 female refugee survivors of torture from 30 different countries suggests that “being female increases the risk of PTSD through a direct increase of cumulative traumatogenic dynamics, stressors that predispose an individual to respond differently to new traumas” (Dehghan, 2018, p. 79). He illustrates gender discrimination (GD) as a type of continuous and systemic trauma that is directly related to identity development. Ongoing gender discrimination in family and society can render a woman more susceptible to the detrimental effects of other identity related traumas, such as poverty, racism, and sexual violence (Kira, 2010, p. 304).

While PTSD dominates the field of trauma studies, the concept has also come under criticism. Specifically, the cause-and-effect notion has been criticized by transcultural psychiatrists for its heavy reliance on a biomedical perspective based on a Eurocentric approach to mental health that can be problematic and lack sensitivity in different cultural contexts (Kirmayer, 2006; Kleinman, 1987; Littlewood, 1996; Summerfield, 2004). In part, this critique refers to what Arthur Kleinman (1977) calls ‘category fallacy,’ the assumption that Western nosological classifications of mental health disorders can be decoupled from their context without losing validity. The impact of and rehabilitation from a traumatic event such as torture involves a re-making of meaning and re-examining of beliefs and values in one’s life, a process that is closely linked to a person’s socio-cultural background (Bracken, 2001; Summerfield, 1999). Cultural validity is about ensuring that an instrument measures what it says it measures with reference to truthfulness of a theory in a particular culture. This is not something researchers can achieve through a mere translation of a questionnaire. There are terms and concepts that may not exist cross-culturally. Distress idioms are culturally loaded and local (Jadhav, 2009), which means that one can be traumatized without having the symptoms typical of a PTSD diagnosis as much as one can be misdiagnosed with PTSD (Table 1).

**Table 1.** Characteristics of the participants.

	Background	Gender	Ethnicity (self-identified)
**Interviewees**			
P1	HCP	M	White British
P2	HCP	F	White British
P3	HCP	F	Irish
P4	HCP	F	Turkish
P5	HCP	F	White British
P6	HCP	F	White other
P7	HCP	F	Turkish
P8	HCP	F	White British
S1	Torture survivor	M	Kurdish from Turkey
S2	Torture survivor	F	Kurdish from Turkey
S3	Torture survivor	F	Kurdish from Turkey
**Focus group participants**			
F1	HCP	F	White British
F2	HCP	F	White British
F3	HCP	F	Turkish
F4	HCP	M	Asian other

HCP: Healthcare Professional; F: Female; M: Male.

The infliction of suffering and its impacts depend on numerous socio-economic and identity markers such as gender, sexuality, class, race, culture, and religion. While gender has been increasingly recognized as a crucial determinant of health, and there is evidence for a connection between gender and poor mental health among refugee survivors of sexual torture, gender has been largely ignored in the torture literature (Bogic et al., 2015; Hooberman et al., 2007; Oosterhoff et al., 2004; Pérez-Sales & Zraly, 2018; Song et al., 2014).

Individuals are born into social systems that manage them as biological and psychological entities based on normative understandings of gender and sexuality. Gender inequalities and power dynamics are entangled in individual experience (Bracken, 2001; Kira et al., 2010; Leatherman, 2011; Quiroga & Jaranson, 2008), and both gender and sexuality are important analytical tools. We conceptualize sexuality as an element of social life (Weeks, 2003). Similarly, gender is not merely an identity marker but is also a part of systemic structures that place people, relationships, and human activities in hierarchical categories (Mazurana & Proctor, 2013). Put differently, “gender is a set of discourses that represent, construct, change and enforce meaning” (Sjoberg, 2007, p. 84).

Taking a feminist perspective that accounts for intersectionality (Crenshaw, 1991) should therefore be fundamental to an investigation of sexual torture. Intersectionality denotes that an individual’s experience is shaped at the crossroads of their position in multiple identities, each of which may mediate the consequences of trauma. In addition, postmodern social theories propose that subjectivity and identity can be described as multiple and constructed (Lancaster & Di Leonardo, 1997), explaining how power and violence enact through practices of gendering and helping us to acknowledge the close links between the infliction of suffering and the corporeality of the victims. Our use of postcolonial feminism consists mostly in the challenge this tradition poses to the liberalist notion of the universal ‘woman’ and ‘man’ and a commitment to the heterogeneity of experience and diversity of agencies that are informed by socio-political locations (Lewis & Mills, 2003; Mendoza, 2016, pp. 107–111). We will use the term ‘conceptual self’ to refer to “socially constructed schema and categories that define the self, other people, and typical interactions with the surrounding world” (Conway, 2005, p. 597). We will also deploy the concept of ‘adaptive responses’ to avoid pathologizing or unnecessarily gendering the diverse array of psychological processes implicated in responses to violence and trauma (Conlin, 2017). The concept of ‘self-conscious emotions’ will be drawn upon to echo the reflective evaluation of oneself (Tangney et al., 2007).

The aim of this qualitative study was to examine the impact of sexual torture by employing a gender-critical perspective; specifically, to investigate how gender mediates the operationalization and psychological impact of sexual torture in Iranian, Afghan, and Kurdish refugees in the UK, paying special attention to drivers of the impact of sexual torture. A series of identity qualifiers that modify the effect of sexual violence in Afghan, Iranian, and Kurdish torture survivors will also be discussed.

The choice of study population calls for explanation, given the challenges of the recruitment of a study cohort for such a sensitive topic (Dehghan & Wilson, 2019). Afghan, Iranian, and Kurdish individuals comprise the largest number of clients from Muslim-majority countries attending FFT. Kurds have repeatedly constituted the top population of referrals to FFT since 1989, with Iran and Afghanistan as the top two countries of torture victim provenance (Freedom from Torture, 2019). These three population groups are heterogeneous in their historical, social, and political positions, given the political instability and ongoing fighting in Afghanistan and the persecution of the Kurdish people in Kurdistan, a geopolitical region spread over Iran, Iraq, Syria, and Turkey.

## Methods

### Study design

This exploratory study sought to investigate the diverse range of issues connected to sexual torture. Face-to-face semi-structured interviews were undertaken to understand the perspectives of torture survivors and healthcare professionals supporting or encountering survivors. This method is widely agreed to be appropriate when exploring sensitive issues about which little is known (Bowling, 2002). Findings were discussed with a focus group of mental health professionals with relevant expertise to confirm findings from individual interviews, to make sense of the data through multiple perspectives, and to reduce potential limitations from researcher bias (Mark et al., 1999).

### Setting and sampling

The study was conducted in collaboration with two specialist centers for torture survivors in the UK, Freedom from Torture (FFT) and the Helen Bamber Foundation. These are two non-governmental medical-humanitarian organizations providing care and rehabilitation for UK torture victims. Professionals from NHS and voluntary mental health community services in London were also invited (mainly by email) to take part in this study.

The specialist centers did not agree to recruit torture survivors amongst their clients. The centers expressed concerns about the vulnerability of torture survivors, the sensitivity of the topic, and the risk of re-traumatization. They also reported they did not know whether any of their previous clients were politically active at the moment (see recruitment criteria below). Purposive sampling was therefore considered the most realistic method for this study.^1^

The principal researcher had worked with torture survivors as patients in her role as a physician as well as a medico-legal report writer at FFT. Therefore, we used snowball sampling as a way of recruiting this hard-to-reach population. As such, indigenous field-worker sampling and facility-based sampling were chosen as the most appropriate techniques (Shaghaghi et al., 2011). Potential participants from amongst torture survivors were contacted through a trusted friend (and survivor himself),^2^ and individuals then decided whether they wished to communicate with the researcher. Upon them initiating contact, an information leaflet was shared with them and an initial phone or face-to-face meeting was arranged. It is important to note that the indigenous field worker exercised well-informed judgment to protect the psychological wellbeing of potential participants and only those individuals who were open about their torture and functional in personal, professional, and political life were approached. In addition, the primary researcher (a clinician experienced in working with torture victims) used the initial phone interaction to informally assess and confirm the self-reported psychological stability and eligibility of participants. In that meeting, the distress pathway was also discussed.

Inclusion criteria for participation in the study by torture survivors were set as follows: 1) aged ≥18 years; 2) history of torture ;3) mentally and physically able to participate; 4) activist, spokesperson, or representative of torture victims (this criterion was used because of evidence about the resilience of torture survivors with a background in political activism (Parker, 1999)). Inclusion criteria for medical professionals for both interviews and focus groups were: 1) current and former doctors and therapists; and 2) experience working with torture survivors from Kurdish, Iranian, and Afghani backgrounds.

### Interview and focus group procedure

A topic guide for the interviews was generated using a literature review related to sexual torture, as well as knowledge from the first author’s (RD) past experience of working in a torture survivor specialist center. The topic guide was revised after consulting with three other experts: 1) the co-author (CO), who is a senior anthropologist and a feminist scholar; 2) an experienced and practicing psychotherapist; and 3) a psychiatrist with extensive experience in treating torture survivors. Open-ended questions were used to collect information about participants’ definition of sexual torture, its psychological health impacts, coping strategies, and mediators of impact, with a special focus on gender and culture. Interviews (45–60 min) were conducted in English, recorded, and transcribed verbatim. After completing data collection, all professionals who had taken part or expressed interest in the research were invited to participate in a focus group.

### Ethics

Ethical approval for the project was obtained from the University College London and FFT’s Research Ethics Committee. They requested that only professionals from FFT, not clients, take part in the study.

### Data analysis

Interview transcripts were imported into NVIVO V.8 for thematic analysis (RD). After coding and merging categories of data into higher categories, the main themes and categories were discussed in a focus group with four experts in the field. The theoretical framework of intersectionality and postcolonial feminism was applied to the interpretation of the data. To increase internal validity, three transcripts were independently examined by CO. The two authors cooperatively interpreted the data.

### Findings

A total of 13 individuals took part in face-to-face interviews and the focus group. Eight healthcare professionals (therapists: n = 5, doctors: n = 3) and three torture survivors participated in interviews. All three torture survivors were Kurds from Turkey. Two of the eight professionals considered themselves to be Turkish. A majority of the professionals (n = 5) were recruited from specialist centers. Sample characteristics are described in Table 1.

Four experts (two from community centers and two from specialist centers) took part in a two-hour focus group where the key findings of the study were discussed. Two focus group members were also interviewed in the first part of the research. Focus group member characteristics are described in Table 2. To preserve anonymity, limited demographic information is provided.

Five general themes were identified: 1) conceptualization of sexual torture; 2) operationalization of sexual torture; 3) impacts of sexual torture; 4) drivers of the impacts; and 5) disclosure and service needs. This article presents findings on the first four topics. Due to the extensiveness of the collected data, a future article will report on service utilization or disclosure.

Themes will be summarized and discussed within a feminist framework. We will first discuss sexual torture definitions before exploring mechanisms underlying the operation of sexual torture and presenting and interpreting the findings on impact and the drivers of impact. It is important to note that most professionals viewed their speaking on behalf of survivors as problematic. Most made repeated and explicit acknowledgement that their comments should be taken with caution since they were simply reflecting on what they perceived as relevant, and they could not know for certain their clients’ experiences.

### Conceptualization of sexual torture

Clear definitions of sexual torture were used by health professionals but not survivors. All eight professionals acknowledged that sexual torture is inseparable from the body. They also stressed that sexual torture – in the experience of victims – was inseparable from torture.

Interviewees recognized the physicality and sheer materiality of sexual torture as an act of violence against the body. A majority perceived sexual violence as a complex and elusive force. One participant expressed it as follows:

Sexuality is such a complex cultural thing … it’s so layered in all our lives, all our cultures. So when … that’s laid bare by something that has happened to you, all those different layers of complexities … seem to have an impact, but I think it’s also just that physical invasion. (P5)

P5’s insight highlights how sexual torture touches upon physical violence as well as upon something less easily definable. These characteristics were also present in a personal experience shared in the focus group by F4: an electric shock was placed on the fingers and then on the genitals. Both forms of torture were described as equally agonizing, but there was something about the invasion of the genitals in particular that felt specifically ‘unacceptable.’

While rape was recognized by participants as the classic form of sexual violence, touching, groping, and physical violence to the genitals were also described. The latter were particularly distressing since they were perceived as a direct sexual threat, a forewarning of the rape that may follow: “You are always waiting to be raped. And that is abusing you mentally, even when you are alone,” S2 explained.

Similarly, non-physical acts that nevertheless involved humiliation of the body—such as urinating on someone or making sexual taunts—were also defined as sexual torture. Victims’ gender also influenced what was considered sexual. S1 regarded the beating of the genitals as part of general torture. In contrast, S3 highlighted that for some female prisoners, simply being naked in front of strange men was as devastating as any overt form of sexual violence. Furthermore, S3 also stressed that while torture and sexual torture were often an inseparable experiences at the time of the event, each act may be differentiated upon further elaboration later in the life of the victim.

### Operationalization of sexual torture

This section presents data explaining the mechanism through which sexual torture enacts as a tool of power and violence. Participants’ understanding of the operationalization of sexual torture can be divided into three inter-related themes: a) the ‘rape-able’ body, b) male rape and the un-making of the man, and c) the ‘immoral’ woman deserving rape.

#### The ‘rape-able’ body: The imaginable versus the unthinkable

Rape was described as ‘imaginable’ for women, but ‘unthinkable’ for men. Two respondents put it the following way:

Men might have been or might have imagined themselves being in physical fights where they got physically hurt, but would never imagine that they would be raped. Women, they might be romantic about sex, but they might have imagined there was something like that. (P2)

When they [men] gather together, when they’re talking about a woman that they dislike very much, they talk about punishing that woman. Quite often punishment, if they’re talking about a man, it’d be “I beat him up,” or “I dry him up,” whatever it is, but with a woman, it’s like “I will shag her, I will fuck her.” (S1)

Rape was linked with the image of the female—rather than male—body. It was indeed proffered as a female-specific punishment by many participants.

#### Male rape and the un-making of the man

All HCPs acknowledged a close link between sexual violence, sexuality, and masculinity. P5 said:

I think for men particularly it’s, “I can completely dominate you, I can do whatever I like to you,” whereas—it’s wrong to say women expect that, I don’t mean that—you know just physically, men can overpower women physically.

Male-on-male rape is described here as an ultimate act of physical submission to and domination by another man. F3 supported this account, adding that while perpetrators often try to confuse the sense of sexuality of their victims by suggesting their sexual arousal, the survivors could instead recognize the power dynamics in play: “I have had a few discussions with [male] clients who had been raped by men. They know it doesn’t mean they are gay. They understand it was not for pleasure but for control and power.”

#### The ‘immoral’ woman deserving rape

Therapists and survivors observed that misogynistic rhetoric around promiscuity or the ‘the whore’ image were evoked to validate rape in women. S2, a Kurdish female activist, recalled: “When I was first detained, I was 17 years old, the first thing I was told was: ‘You have slept with too many men, and we’ll also rape you.’” P2, recounting the experience of one of her clients in an Iranian prison, suggested that degrading women might be a tool to dissolve psychological barriers toward perpetrating violence against women. She said:

… telling her [Iranian activist]: “You’re a street woman. You’re wandering around, so we picked you up. Now we do whatever we want to you because that’s what you want” … “we know you’re not married, you’re a prostitute, that’s why you’re not a virgin.”

The female prisoner is here being pushed to believe that she was singled out for being a ‘bad woman’ and accused of promiscuity—a claim that is linked with her physical mobility and political activity in ‘wandering around.’

### Impact of sexual torture: Adaptive emotional and psychosocial responses

As noted above, PTSD is commonly associated with torture. However, study participants noted that responses often varied between individuals. While a majority of the professionals believed that there were no distinctive features of sexual torture that would differentiate its impact from torture in general, they also asserted that sexual violence often impacted the intensity, chronicity, and intractability of the psychological consequences in survivors. In P5’s words: “it [sexual violence] often explains the level of psychological symptoms and distress that a person is experiencing.”

In line with the ‘conceptual self’ framework, consequences of sexual torture were divided into five categories: 1) impact on bodily self, 2) impact on self-conscious emotions, 3) impact on inter-personal relationships, 4) changed socio-political consciousness, 5) denial as a distinct response to sexual torture.

#### Impact on bodily self

The sexually violated body was described by interviewees as a source of pain and suffering. Victims perceived their bodies as ‘dirty,’ obsessively washing, hiding, or becoming detached from it. That said, body rejection was not an inevitable response. For instance, S3, a Kurdish activist, described how she stopped hiding her body following sexual torture by consciously shifting the perception and treatment of her body to incorporate a feminist response and resistance to victimization:

Because I read about feminism in prison … So, it helped me to understand my body, my connection with it, my beliefs, and the place I take within the society. So after that, I felt more liberated, because I thought they were trying to take control of my body by shaming my body, but if I didn’t have to hide, if I didn’t feel shame because of my body, they wouldn’t have any control over me. (S3)

#### Impact on self-conscious emotions

Guilt and shame were reported by all participants as common emotional reactions to sexual torture. Some interviewees thought that women were more likely to feel guilty and blame themselves, while men, P6 thought, ‘blamed out.’ She stated that she sometimes recognizes shame disguised as arrogance or anger in her male clients. With regard to shame, while some respondents stated that it was more common in men, others thought that it was more common in women; the majority thought it applied equally.

#### Impact on interpersonal relationships

An overriding finding was that sexual torture impacted “relationships in the widest possible sense” (P3). On the whole, a generalized anger, especially mistrust of people in positions of authority, was often reported. Sexual torture implicates the social and public self in a number of other ways. A sexually violated woman is often seen as unsuitable for mothering in the eyes of others—and sometimes even by herself. P2 recounted how an Iranian client was threatened by her husband’s family with death and loss of custody of her child after they found out that she had been raped in prison.

More specially, participants observed that women may lose social attractiveness following sexual torture. When feminine desirability is attached to virginity, raped women lose their marriageability. Female political activists are likely to retain their political status but still lose their social positioning as a marriageable woman. Some women may still consciously choose domesticity in order to re-establish their social place as a ‘womanly’ woman. This was most poignantly conveyed by S3:

People in society knew that if someone was in prison, they must have been sexually harassed, so … although they respected them politically, they thought those women were already ruined. No married life after prison, maybe. And some of my friends had to go back to a traditional life after prison because they wanted to prove [to] the society that they are still women.

In addition, secrecy, silence, anger, and especially shame were reported as impacting sexual intimacy. S3, reflecting on her female acquaintances and friends, reported that some women may turn to sex as a form of retaliation or expression of their repugnance of intimacy:

Some of them [female survivors] … crave sex … Not that they feel helpless, they want to use their sexuality as a revenge. They don’t want long-term relationships, only short-term … I think they hate sexuality, not men. They just hate intimacy.

Interviewees commented on how sexual violence inhibits sexuality as a source of pleasure. Instead, disgust of sexual intimacy, of one’s own body, and of any form of physical contact with others becomes pervasive. Lastly, sexual torture can lead to social isolation. The most striking reason for social isolation was a belief that one is the harborer of ‘bad luck.’ Survivors may avoid closeness with others in order to protect them as “they feel they bring something bad to others” (P6).

#### Impact on (socio-political) consciousness

Besides their political identity, the two female survivor-participants both referred to the gendered aspect of themselves. They reported having become more conscious of gender discrimination in society at large and becoming interested in advocacy for women’s rights. S2 summarized the social impact of sexual torture as follows:

You become a feminist [she laughs] because you think you are not the only person subjected to this issue. You realize there are too many women, in fact, even men, having those kinds of problems. You become interested in this issue and want to do something about it.

#### Denial

Denial and non-disclosure, especially among men, emerged as specific mechanisms used to respond to sexual torture. Some HCPs thought this resulted from exposure anxiety or the emotional distress of speaking about it, whereas others conceived it as a deliberate silence and valid coping strategy.

### Drivers of impact

We have placed the drivers of impact into three categories: 1) trauma-related, 2) identity markers, and 3) factors related to group identity. This categorical separation is merely formal: lines between them are fluid and they are mutually dependent. When first asked, most participants minimized the contextuality of sexual torture. Nonetheless, during interviews, all provided accounts that to some degree contradicted that initial response.

#### Trauma-related factors

All participants agreed that sexual torture tactics that make use of psychological factors to inflict suffering are the most enduring. All types of sexual torture, including threat of rape, forced nakedness, verbal slurs, and vulgar language, were noted as tormenting. S3, for example, recounted: “Some female friends who were with me in prison, they were devastated only because they had to get naked in front of the police.” Oral penetration was also mentioned as a specific and distressing deviation from cultural norms. Still, all participants, without exception, underscored rape (both anal and vaginal) as the key marker of sexual violence standing out in its impact on the victim. Furthermore, violating a victim in a family member’s presence increases the sense of vulnerability and helplessness as well as guilt, blame, and shame.

Time was noted as another factor mediating impact, since psychosocial responses are likely to shift over time. Such changes may represent positive or negative associations. The initial anger that helped a victim cope in the immediate aftermath of sexual torture may diminish with the passage of time. Time may provide an opportunity for re-evaluation and meaning-making, potentially supporting recovery.

#### Identity markers

Personality traits, age, sexuality, and religion were listed as individual factors driving the experience of sexual torture. Resilience was conceptualized as a personality trait by three professionals. Therapists from minority backgrounds and survivors mostly presented images of resilience, resolve, and agency as linked to socio-political and cultural aspects of being-in-the-world rather than personal traits.

The impact of age was often explained through other mediating factors such as the existence of family support or the presence or absence of previous sexual experience. However, the direction of the association remained inconclusive.

The notion of virginity as an important determinant of the impact of sexual torture was brought up by a majority of participants. The cultural significance of virginity means that threatened or actual loss of virginity, as well as nakedness, can be devastating experiences for some women. What constitutes trauma is as much socially constructed as is the response to ‘trauma,’ as illustrated by this excerpt:

I think the loss of virginity can be for some women an additional huge loss, also potentially a life-destroying thing … I mean in women who’ve already perhaps been sexually active up to the point of the sexual violence, and who’re also in a country where that [not being a virgin] is okay, they have all the [other physical and psychological] problems [too], but at least there’s one problem they don’t have. They don’t have the problem of loss of virginity and what that means for their future. (P8)

One striking finding came from the interview with S3, a participant with extensive experience as a therapist in a sexual health clinic in London. She disclosed that her clients who were gay male survivors of sexual torture from Iran experienced an extreme sense of isolation—resulting partly from dual trauma of pervasive homophobia in the Iranian community and from racism in the gay community in London.

With regard to religion, most professionals asserted that, in contrast to other clients who found strong support in religion, their Kurdish and Iranian clients were either mostly not religious or did not speak of religion in a beneficial way (when the divine was perceived as retributive or silent in the face of cruelty). Subversion of cultural and religious norms brought about by the experience of sexual torture may create confusion by disrupting one’s world-guiding principles. A small number of respondents, reflecting on their Iranian clients, observed that religion may be lost as a source of personal support when perpetrators and authorities carry out sexual torture ‘in the name of religion.’

#### Factors related to group identity

Political identity, family and community, gender, as well as ethnicity and nationality were amongst the group identities mediating the impact. All three Kurdish survivors were vocal about their political (as well as gender) identity being the main motivator for their participation in this study. Throughout the interviews, they often employed a collective frame of reference in their trauma narratives. This seemed important in creating meaning and explaining the suffering that was inflicted upon them. This echoed the experience of P3, who described how a Kurdish colleague-survivor would externalize his experience by saying: ‘the shame is not mine.’ This framing alters the experience of suffering.

Political identity is one commonality used to create community, though this does not necessarily hold true for all national and ethnic groups, genders, or sexual orientations. For example, P4 noted that Afghan and Iranian women were less likely to engage in the community. P7 also commented that “[a] woman can become more isolated, more detached from community because there are cultural issues.” P1 also recalled the conflict of an Afghan woman who felt she had to accept her abusive husband in order not to be shunned by her community.

The importance of family and social support was also endorsed by participants in the focus group. The fear of rejection and blame by one’s community can result in distress, non-disclosure of sexual torture, loneliness, and isolation in the host country. P3 thought that this was most poignantly expressed in the case of gay Iranian refugees: “Before you even add in their experience of violence and torture, feeling ‘Where do I fit?’, ‘Where do I belong?’”

Respondents observed that female survivors of sexual torture were more likely to feel disgust toward men in general. They agreed that male against male rape was generally a taboo topic that may potentially lead to non-disclosure as well as to severe psychological distress and isolation. Making therapy a viable option for men may, they felt, be more challenging. In the focus group, F2 pointed out that “women in every culture get support to disclose, but for men it is more difficult.”

Interestingly, most survivors and therapists asserted that gender discrimination and general everyday hardships are life themes for women in a way that doesn’t exist for men; hence, sexual assault presents a different shock to a man than to a woman.

Lastly, the findings suggest that the historical and political landscape in one’s country of origin influences a survivor’s experience. For instance, P3 noted that her African patients did not have a strong political identity, unlike her Iranian and Kurdish patients. Participants in the focus group also acknowledged that their Afghan clients are mostly younger men, a characteristic linked to the country’s enduring conflicts.

## Discussion

The findings from this interview and focus group study are consistent with evidence from previous research and clinical work that survivors themselves make no formal distinction between sexual and non-sexual torture. What constitutes sexual torture emerged as fluid, temporal, and contingent on the gender of the victim and of the perpetrator, the act, the sexual meaning attached to the act, the role of the body, and the attitude and belief systems of all actors. Most participants’ conceptualization of sexual torture corresponded with the current definition in the field that encompasses violence directed at sexual organs and physical and psychological sexual assault, including sexually charged insults and gender-based attacks (Agger, 1989; Lunde & Ortmann, 1990; Pérez-Sales & Zraly, 2018).

It is fair to say that sexual torture is inseparable from wider discourses on the body, sexuality, and gender (Sansani, 2004). As Puar (2005) noted and as Campbell (2018) insists, there is nothing exceptional or singular about sexual torture practices; these acts live within a continuum of socially acceptable and norm-constituted expectations of gendered behavior. In fact, the power of an action, discourse, or gendered performance lies in the reiteration and citation of the dominant narrative (Narkassis, 2013). The body–sexuality dynamic was highlighted by approximately half of the respondents, especially in the context of rape. The nexus of embodiment, gendered body parts, and gendered acts appears throughout our material, revealing sexual torture as having efficacy exactly because it works through exaggerated hyper-examples of gendered dynamics discernible in everyday life, as noted in the Gender Justice framework (Campbell, 2018, p. 484). When the act of penetration has been so successfully coded as masculine, the gendered effects are palpable (Butler, 1993; Selgas, 2014).

A distinction emerged between rape as ‘imaginable’ for female bodies and ‘unthinkable’ for male bodies, while penetration was perceived as an element of the ‘imaginary body’ of the woman. The ‘imaginary body’ is a concept that explains how “power takes hold of and constructs bodies in particular ways” (Price & Shildrick, 1999, p. 230). By committing an act that establishes dominance, the torturer seeks to display absolute physical control over the male victim, stigmatizing him as subordinate, feminized, or completely emasculated. Physical power, penetrative capacity, heterosexuality, and the capacity to reproduce all act as symbols of masculinity. Since masculinity asserts and protects itself in relation to femininity through violence, it is vulnerable to destruction and contradictions (Loncar et al., 2010; Sivakumaran, 2007).

As for the woman, notions of subordination, objectification, and immanence are key to sexual torture. Rape is widely coded as a woman-specific punishment and the subjugation of women is replicated and accentuated through sexual torture (Campbell, 2018, p. 484). Cultural narratives create binary formulations of ‘the decent woman’ versus ‘the whore’ and the ‘moral’/‘immoral’ dichotomy. These divisions are closely linked to supposed or actual sexual conduct as well as to distinctions between the feminine domestic sphere and the masculine public space. Once a woman is judged immoral and her body impure, socio-cultural norms protecting the ‘respectable’ woman are no longer extended to her. Our findings mostly support Agger’s (1989) psychodynamic analysis of the operation of sexual torture. She rightly maintains that the aim of sexual torture is “to deprive the victim of his or her identity” (Agger, 1989, p. 307). As such, the impacts of sexual torture are as broad as they are unique to each victim.

While a wide range of psychosocial effects of sexual torture such as PTSD, dissociation, depression, anxiety, suicidality, sexual dysfunction, personality changes, and social withdrawal have been frequently documented in the literature (Isakson and Jurkovic, 2013; Peel, 2004), we identified five specific categories affecting survivors: impact on bodily self, impact on self-conscious emotions, impact on inter-personal relationships, a changed (socio-political) consciousness, and denial. For a victim of torture, “the body in itself becomes a source of threat” (Ataria, 2016, p. 9); “When body is identified with trauma, it is disowned” (Ataria, 2016, p. 6). Disgust around the body and sexual activity, as well as changes to self-image, are frequently described in our study. Unsurprisingly, shame was the most cited emotion. Shame, defined as “a sense of worthlessness and powerlessness” (Tangney et al., 2007, p. 349), often appears after moral injury (perceived violation of the principles of decency), especially when events disrupt the ‘social order’ or “remind us of our animal nature” (Tangney et al., 2007, p. 361). Lee et al. (2001) argue that if one does not identify with the shaming, then the response may be anger and a sense of humiliation. Correspondingly, blame occurs when responsibility for the violation is externalized, often connecting the harmful conduct with the actual perpetrator.

Another anticipated finding was the negative impact sexual torture has on all forms of relationships, from close family members to distant acquaintances. Sexual torture threatens the whole social fabric of the victim, undermining the respectability of the political self and confidence in one’s ability to meet expected gender norms. Further, feminine desirability is attached to virginity; its loss undermines marriageability and “can constitute a threat to the entire public life project of a woman” ((Pérez-Sales & Zraly, 2018, p. 6). As bodies exist in social contexts, a female survivor can transcend her confinement to the body through either political activism or marriage, reclaiming the social status that sexual violence threatened to diminish. Marriage as a social norm and a moral act abates the deviant and immoral embodiment of rape on a woman’s body, restoring a woman’s body back to society. Some women, in order to resist their objectification, subvert gendered cultural practices by turning to casual sexual relationships. Sexuality becomes a transgressive site, a source of counter-discourse, minimizing the act’s effects by reframing cultural norms of modesty. Alternatively, such actions could represent another form of loathing of body and sexuality. Only case-by-case analysis can unravel the complex and often mixed motivations behind the actions and reactions of individuals.

In terms of the mediators of impact, our results are consistent with a growing body of evidence that the mental health impact of torture is shaped by “perceived distress and controllability of torture stressors, not just exposure to them” (Quiroga, 2008, p. 3). Dominant cultural narratives influence the formulation of subjecthood and meanings attached to the body. It is one of the modalities that helps us organize experience and assign importance to it (Peel, 2004; Pérez-Sales & Zraly, 2018).

We classified mediators as either trauma-related or connected to various ‘individual’ or group identity markers. The participants from minority ethnic groups endorsed a more situational and contextual framework rather than a personality trait approach. The geo-political context of trauma was also stressed. Younger, mostly male Afghans coming from war zones are more inclined to religious frameworks for meaning-making than Kurdish and Iranians who often experienced torture in a prison setting. The type of sexual torture impacts the response as well. For example, anal or oral penetration may be regarded as particularly shameful for women. Time elapsed since trauma is another key factor: torture survivors who evaluate and reflect upon their experiences will, over time, develop new phenomenological understandings of their position in the world.

The effect of individual factors such as personality, age, and religion was inconclusive (Isakson & Jurkovic, 2013). As the authors of *Crime and Impunity* (Sadr & Amin, 2012) note, this study also observes that women who hold strong beliefs about sexuality or are sexually inexperienced may feel distressed in a different way from those who do not. For women, a key variable was the individual and social significance of virginity as “the hallmark of femininity” (Loncar et al., 2010, p. 270) and the impact of its loss on “marriageability.” We note that this concern was also expressed by Iraqi women in a study investigating consequences of sexual torture under Saddam Hossein’s regime. In fact, many of those women never married (Einolf, 2018).

Following sexual torture, the respectability of a woman in her political role may mitigate her diminished gender status. However, this also cuts the other way; an apolitical woman who has been raped in order to punish her family may not benefit from the buffer of a political identity. Other forms of supportive group identities which moderate the impact of trauma can be forged around ethnicity, a phenomenon most pronounced amongst Kurdish survivors.

Yet, accounts that refer to communities as ‘resilience asset(s)’ (Alemi et al., 2016) fail to consider how community members relate to each other based on particular norms and expectations, the transgression of which leads to exclusion. Ethnic group identities are modulated via sexuality and gender. The most revealing finding was the enormous isolation of Iranian gay survivors, due to homophobia in Iranian communities on the one hand and racism in the gay community on the other hand. This issue merits further research. Ethnic social support is found to be strongest for male (presumably heterosexual) refugees rather than for other demographics. What is more, living up to the culturally assigned role of breadwinner and protector may be crucial to the social status and self-worth of a man (undoing the unmaking of masculinity). There is some evidence in the general corpus of torture literature suggesting that the importance of employment as a predictor of mental health outcome is gendered. Male refugees in general suffer more from ‘downward mobility’ (Young & Chan, 2015). A study of torture survivors in Denmark confirms poor social support and unemployment as main factors associated with emotional distress (Carlsson et al., 2006).

In terms of group identity, it was striking to discover that the recognition of oppression may become a source of resilience for women. Their trauma can be understood as part of a coherent narrative about violence against women, helping them develop a stronger gender identity and integrate the personal with the political, contextualizing the extreme experience.

Lastly, our respondents reported that religion did not play a key role for their Iranian and Kurdish torture survivors, unlike for Afghans, as empirical literature suggests that social support and religion are the key coping strategies for mental health issues amongst Afghani refugees (Alemi et al., 2016). However, there remains a lack of research into the association between religion and wellbeing for torture survivors from Muslim backgrounds.

### Limitations

This study has a number of limitations. For ethical reasons, only a small number of Kurdish torture survivors were interviewed, with no direct accounts from Afghani and Iranian communities. The small sample size and the use of snowball sampling in recruitment of Kurdish survivors, who were all politically active, limit the generalizability of the findings. In addition, most data were indirect accounts from professionals based in London rather than victims. However, access to victim interview material conducted with professionals provided some first-person material. We acknowledge that the accounts from professionals were shaped by their specific positionality and investment in the dominant discourse. This potential bias is commonly noted within related fields (Lancaster et al., 2017; Porter, 2018). Although we incorporated member-checking and independent coding to increase reliability and validity, we are aware the views of researchers have also shaped the interpretation of the data. It is important, therefore, to note that our data analysis provides only an initial understanding of the experience of this particular cohort and the specific characteristics of each community and individual will determine the impact of sexual torture.

## Conclusion

This study applied a gender-critical perspective to understand the experience of sexual torture directly for Kurdish survivors in London and indirectly for Afghani, Iranian, and Kurdish migrants in the UK by drawing on the clinical expertise of therapists and doctors in two specialist centers for torture survivors as well as in community mental health services. We examined the conceptualization and operation of sexual torture as well as its psychological health impacts and the mediators of such impacts. It seems plausible to say that sexual torture leverages the dominant discourse and operates through a reiteration of hegemonic norms. It also impacts the bodily self, self-conscious emotions, and inter-personal relationships and changes (socio-political) consciousness. Denial may be a conscious response to sexual torture, too. The impact is mediated by variables that are related to the trauma itself, and to those associated with individual or group identity. Recognizing these dynamics helps to reconcile the sometimes differing and conflicting modes of interpretation of patients and to improve healthcare professionals’ understanding. Recognition and validation of victims’ experiences by professionals is an important step toward rehabilitation. It is recommended that future studies take into account the legacy of coloniality and its ongoing intersection with trauma in their research question and methodology.

## Notes

1. The ethics of gatekeeping was examined by RD in Dehghan and Wilson (2019).

2. While contacts in all three communities (Afghan, Iranian, and Kurdish) were approached, only the Kurdish facilitator was successful in recruiting research participants.
